# Laboratory Information Management System Chain of Custody: Reliability and Security

**DOI:** 10.1155/JAMMC/2006/74907

**Published:** 2006

**Authors:** J. J. Tomlinson, W. Elliott-Smith, T. Radosta

**Affiliations:** 1 ChemWare,Inc. 900 Ridgefield Drive Raleigh NC 27609 USA

## Abstract

A chain of custody (COC) is required in many laboratories that handle forensics, drugs of abuse, environmental, clinical, and DNA testing, as well as other laboratories that want to assure reliability of reported results. Maintaining a dependable COC can be laborious, but with the recent establishment of the criteria for electronic records and signatures by US regulatory agencies, laboratory information management systems (LIMSs) are now being developed to fully automate COCs. The extent of automation and of data reliability can vary, and FDA- and EPA-compliant electronic signatures and system security are rare.


Hindawi Publishing Corporation
Journal of Automated Methods and Management in Chemistry
Volume 2006, Article ID 74907, Pages 1–4
DOI 10.1155/JAMMC/2006/74907

### Laboratory Information Management System Chain of Custody: Reliability and Security

**J. J. Tomlinson, W. Elliott-Smith, and T. Radosta**

*ChemWare, Inc., 900 Ridgefield Drive, Raleigh, NC 27609, USA*

Received 18 September 2005; Revised 17 January 2006; Accepted 18 January 2006

A chain of custody (COC) is required in many laboratories that handle forensics, drugs of abuse, environmental, clinical, and DNA testing, as well as other laboratories that want to assure reliability of reported results. Maintaining a dependable COC can be laborious, but with the recent establishment of the criteria for electronic records and signatures by US regulatory agencies, laboratory information management systems (LIMSs) are now being developed to fully automate COCs. The extent of automation and of data reliability can vary, and FDA- and EPA-compliant electronic signatures and system security are rare.

Copyright © 2006 J. J. Tomlinson et al. This is an open access article distributed under the Creative Commons Attribution License, which permits unrestricted use, distribution, and reproduction in any medium, provided the original work is properly cited.

### 1. OVERVIEW

A chain of custody (COC) is the set of traceable records that provide unbroken control over a document, raw data, or a sample and its containers from initial collection to final disposal [1]. It is required in laboratories that handle samples bound by legal or regulatory directives, including those enforced by United States government agencies such as the Department of Transportation (US DOT), the Environmental Protection Agency (US EPA), and the Food and Drug Administration (US FDA). To meet those legal and regulatory requirements, many laboratories are required to keep detailed chain of custody records for all of the samples that move through their facilities.

Historically, COCs comprised volumes of paper documents created and maintained by laboratory and administrative personnel. With laboratory information management system (LIMS) technology development and the establishment of regulatory standards for electronic records, manual systems are being replaced by electronic ones that are created and maintained by LIMS.

Laboratory information management systems with secure, flexible open database connectivity (ODBC) are equipped for the enhancements needed to build a complete, protected electronic tracking system and to maintain custody records. Until recently, all that was needed were the regulations for implementing security.

The principle US regulatory agency criteria for electronic recordsand signatures that were established to fill those needs include the United States Food and Drug Administration's 21 Code of Federal Regulations Part 11 (21 CFR Part 11), released in 1997 [2, 3], and the ensuing United States Environmental Protection Agency's Cross-Media Electronic Reporting and Recordkeeping Rule (the CROMERR Rule). Their establishment enabled the LIMS development needed for, and that has led to, secure electronic record keeping for COC.

Several LIMSs currently provide tools for COC, though the reliability of tracking by those systems and the extent of automation of those systems vary widely.

### 2. TYPES OF CHAINS OF CUSTODY

Laboratory-based chain of custody strives to answer the following questions.

(i) "Where is my sample now?"
(ii) "Who possesses my sample now?"
(iii) "When did he/she take possession of my sample?"
(iv) "Where has my sample been?"
(v) "Who has been in possession of my sample?"

In a LIMS, chains of custody must be based on some entity that all of those questions' answers can tie into. It must retrieve information about that entity reliably and always be user recognizable.

Most LIMSs ultimately use a combination of entities, but each uses one entity as its base of reference. And each entity has its benefits and limitations.

### *2.1. Sample-based chain of custody*

The most common entity that electronic COCs are based on is the sample. At sample login, the COC starts and tracks the sample through all procedures until its disposal.

In high throughput laboratories, samples may be split up and used in multiple processes. When split and dispersed to different processes and locations at different times, confusion about the branch in a sample's COC often arises. Because of this, users cannot accurately answer any of the COC questions and this is not a true chain of custody.

Sample tracking can also overlook these important components of laboratory work: standards, quality controls, and solutions. Sample-based identifiers can be modified for assignment to these different entities, but tracking can remain confusing. Identifiers must compensate for the differences between samples and other entities and system workflow must be altered to allow solutions, standards, and controls to enter the COC at different times while forbidding samples the same flexibility. A wider COC basis is preferred.

### *2.2. Location-based chain of custody (radio frequency identification)*

Location-based COCs use radio frequency identification (RFID) to track containers affixed with labels that have embedded microchips and antennae. RFID readers track samples in and between given locations. Each location's readers have distinct identities and these readers monitor the locations of samples. This system can locate a container and its label anywhere within reach of the RFID reader and can read labels that are contaminated or obscured [4]. The questions about "where" a sample is can be answered reliably. However, the "who" questions cannot be assured.

Furthermore, the reliability of the reader can depend on its compatibility with the equipment in the room. Interference from instruments such as mass spectrometers, electrochemical detectors, or refrigerators and freezers can interrupt the flow of radio signals and cause reader errors. Additionally, laboratories designed and built to minimize inter- or intra-laboratory instrument interference can prevent location-based RFID readers from locating RFID-labeled containers that move through labs or into adjacent areas. The effort and cost of the backups required to make this configuration reliable, plus the cost of the microchip- and antenna-embedded labels, render this system too costly for budgeted laboratories. Interference from instruments can make it too unreliable for high-throughput laboratories.

### *2.3. Container-based chain of custody*

Container-based COCs can track every type of container that enters the laboratory. Every sample, control, and solution enters the system according to its vessel type and ID, which are directly connected to its contents through the database. Users predefine all possible container types, so any container that enters the lab can enter the COC. Neither time nor mode of entry is a factor.

With container tracking, the sample that is in the container, any procedure it is involved in, the person who has the sample, and its current location are obvious. There is none of the confusion caused by sample overlap seen in sample-based COC systems and all containers can be recorded, no matter what they hold. It requires none of the equipment seen in location-based systems and there is no electronic interference.

The one LIMS with a container-based COC is also equipped with regulatory-compliant electronic signatures. It is the HORIZON LIMS.

### 3. THE CONTAINER-BASED ELECTRONIC CHAIN OF CUSTODY

HORIZON's cradle-to-grave container-based COC can track every container that enters and moves through the laboratory. This includes containers used to collect samples and their spares, distribute sample aliquots, hold controls, standards and stock solutions, perform wet chemistry procedures, conduct final analyses, and verify disposal. All means of handling and processing samples are tracked and monitored. It also uses electronic signatures that comply with the US FDA's 21 CFR Part 11 and the US EPA's CROMERR Rule.

The types of containers the LIMS recognizes are defined by the laboratory and can include any type of container that the laboratory uses. Each has a unique, unalterable ID that ensures its permanent security and has a user-recognizable description. New container types can be added and new container IDs inserted at any time, letting users work through problems easily.

At any time, the LIMS can show details about any container in the system, including its

(i) physical characteristics,
(ii) contents, including volume,
(iii) associated samples,
(iv) current custodian,
(v) past custodians,
(vi) current location,
(vii) past locations,
(viii) reasons for transfers,
(ix) current procedure,
(x) past procedures,
(xi) anticipated disposal date,
(xii) the complete history, even in a screen capture.

A "chain" is the set of container-based electronic documents that make up the COC. To meet regulatory requirements, each chain has a unique ID that is never changed, duplicated, or deleted, ensuring its security.

The "custodian" is the person responsible for the condition of the container and its contents when in possession of it. That person must keep the container in view and possibly put it in a secure location. It is also in custody when stored in a designated secure location [5].

### 3.1. Container entry

Containers enter the laboratory and the LIMS COC at different stages of processing and during different types of lab work. Many containers enter the LIMS when their associated samples are logged in. Multiple containers of different types may be linked to a sample during login depending on the procedures assigned to it.

Containers also enter at logical points during processing, such as when solutions are mixed, sample preparation batches are created, or extracts are transferred to vials for analysis.

### 3.2. Container labels

Labels are printed automatically in text and barcode for all containers that enter the system. The LIMS knows what type of label is required for a particular container type, ID, user, label printer, and label reader. Labels can be preprinted or printed automatically as needed. They can be printed in text, barcode, or other predefined format.

### 3.3. Container volume

Container volume tracking is valuable when needed. The system needs to know the container's original volume, as well as the expected and minimum volumes for a container-analysis combination. Scripts continuously calculate and track the volume and issue user warnings if a problem is anticipated.

### 3.4. Container location

Container locations can be buildings, laboratories, lab areas, or storage units such as freezers, refrigerators, cabinets, shelves, boxes, or racks.

Some locations must be designated "secure" to provide security when inconsistencies in transfer must be permitted to occur, such as in labs with multiple shifts where custodians change between shifts.

### 3.5. Container transfer

Groups of containers often move together on a chain, but when one or more containers must be separated from the others, new chains are created to accommodate them. Such separations can happen often, such as when organics are separated from inorganics during general storage or when samples are batched and prepared together, and then split up to be batched and run through different analyses.

### 3.6. Quality control containers

Control container tracking is critical. The LIMS is prepared to include controls automatically at any logical point during sample processing and analysis; each point is predefined in the LIMS by the laboratory's analytical processes.

Particularly important are the controls used to monitor analytical performance during sample processing and analysis. When control containers are added to a batch of sample containers, those controls are added to the batch's chain.

### 3.7. Solution containers

Solution containers can be accepted at any time. Volumes and concentrations of solutions can be tracked and warnings can be issued when a volume limitation, such as not enough sample for a procedure or too little standard for spiking, is calculated. Spiking solution volume tracking is also used in final results calculation to automatically determine quality control parameters such as percent recovery or relative percent difference.

### 3.8. Container disposal

Container disposal is the last step of the COC. Once a container is disposed of, no other procedures can be performed using that container ID. If the laboratory recycles containers, it is assigned a new ID and used on future chains.

When needed, samples can be disposed of by the LIMS. When a sample undergoes disposal, all of its associated containers are disposed of simultaneously. Its disposal history is stored with the container disposal history, where it becomes part of the COC.

### 4. ELECTRONIC SIGNATURES AND SECURITY

Electronic signatures authenticate the identity of users who accept and release containers during container receipts, transfers, and disposals.

The HORIZON LIMS electronic records and signatures module complies with the US FDA's 21 CFR Part 11 and the US EPA's CROMERR Rule. It uses the user's logon ID and encrypted password as the main signature components.

Validated electronic signatures are required from the custodian who receives the initial sample, by those who relinquish and receive containers during transfer, and those who relinquish and discard containers at disposal.

Electronic security also protects sample work and retention requirements. For instance, at reporting time, the LIMS can warn when outstanding work is scheduled and, when a sample disposal is indicated, the system can warn if the scheduled disposal date has not yet passed.

### 5. REPORTING

To meet the needs of all laboratories and laboratory clients, COCs must be stored and viewed both electronically and in hard copy and with authorization. Therefore, printed reports of all steps described above are generated accurately and when required. Only those specifically authorized to do so can generate these reports.

### 6. CONCLUSION

The container-based fully automated HORIZON chain of custody is the most reliable and complete LIMS tracking system available today. It gives cradle-to-grave tracking of all samples, controls,standards, and solutions and gives details for electronic or hard copy reporting. The system's regulatory compliant electronic signatures add validation unavailable through other LIMS. This system's development and implementation add no additional cost and require no custom interfacing. All components described are a part of the standard LIMS.

### REFERENCES

[1] International Union of Pure Applied Chemistry, "Glossary of Terms Relatings to Pesticides," Accessed June 2, 2005, http://www.iupac.org/reports/1996/6805holland/g1.html #glp_chain.

[2] U.S. Food and Drug Administration, *Federal Register*, vol. 68, no. 37, pp. 8775–8776, 1997.

[3] K. L. Keatley, "A review of US EPA and FDA requirements for electronic records, electronic signatures, and electronic submissions," *Quality Assurance*, vol. 7, no. 2, pp. 77–89, 1999.

[4] M. Venkatesan and Z. Grauer, "Leveraging Radio Frequency Identification (RFID) technology to improve laboratory information management," *American Laboratory*, pp. 11–14, 2004.

[5] Vermont Department of Environmental Conservation's Environmental Laboratory, "Section 7: Sample Management and Chain of Custody Procedures," Accessed July 30, 2005, http://www.anr.state.vt.us/dec/lab/htm/qualitycontrol.htm.

